# PReS-FINAL-2232: Long-term follow-up in a national cohort of MKD patients: search for clinical predictors of a spontaneous improvement

**DOI:** 10.1186/1546-0096-11-S2-P222

**Published:** 2013-12-05

**Authors:** M Doglio, S Federici, A Tommasini, A Meini, M Cattalini, L Obici, L Cantarini, F Zulian, L Breda, R Consolini, F Corona, A Insalaco, D Rigante, A Martini, M Gattorno

**Affiliations:** 1 Pediatria 2, IRCCS ISTITUTO GIANNINA GASLINI, Genoa, Italy; 2 IRCCS Burlo Garofolo, Trieste, Italy; 3 Spedali Civili, Brescia, Italy; 4 Ospedale S. Matteo, Pavia, Italy; 5 Ospedale Santa Maria alle Scotte, Siena, Italy; 6 Salus Pueri, Padova, Italy; 7 Policlinico SS. Annunziata, Chieti, Italy; 8 Stabilimento di Santa Chiara, Pisa, Italy; 9 Ospedale Maggiore Policlinico, Milan, Italy; 10 Ospedale Bambino Gesù, Rome, Italy; 11 Policlinico Gemelli, Rome, Italy

## Introduction

Mevalonate-kinase deficiency (MKD) is an autosomal recessive disease caused by the deficiency of the mevalonate-kinase enzyme (MVK) and characterized by recurrent fever episodes with systemic involvement. Classically, the disease was thought to have a self-limiting course, with a spontaneous improvement in the adulthood. In 2008 the International registry showed that 50% of patients present a reduction of fever episodes in the adulthood. Up to now, it's difficult to predict the course of the disease a patient will display.

## Objectives

To identify clinical predictors of a self-improvement course of MKD.

## Methods

Patients carrying two mutations of the MVK gene or a single mutation with a MKD phenotype were collected in a national multicentric study. Detailed clinical information was collected at the time of molecular analysis and last follow-up through a standardized questionnaire. Spontaneous disease course was classified as follows: i) resolution (no episodes in the last 6 months), ii) improvement (reduction of more then 30% of fever episodes), iii) stationary and iv) worsening (increase frequency of fever episodes or appearance of new major clinical manifestation). A spontaneous improvement was considered a reduction or resolution of fever episodes without any maintenance therapy.

## Results

We collected baseline information of 56 patients (29M and 27F). The mean age of onset was 10,5 ± 15,3 months (range 1-108), with a number of fever episodes per year at the baseline of 13,8 ± 5,4 (range 3-30). The most frequent mutation was V377I, showed by 43 patients; in 10 patients it was at the homozygous state. Follow-up information was available for 42 patients; the mean age of the patients was 13,3 ± 8,5 years, the mean disease duration was 12,4 ± 8,7 years. At the follow-up the mean number of fever per year was 8,8 ± 6,7. Twelve patients (28,6%) showed a spontaneous improvement of the disease at the follow-up, fifteen (35,7%) remained stable and seven (16,7%) worsened. Thirteen patients required a biologic therapy: five patients improved with Anakinra and no one with Etanercept.

The variables associated with a spontaneous improvement were: female sex (p = 0.019), V377I at the homozygous state (p = 0.03). The same patients display also a less frequent presence of some clinical manifestations at the last follow-up, such as exudative pharyngitis (p = 0,03), painful lymph nodes (p =0,02) and vomiting (p = 0,03). Multivariate analysis indicated as predictors of spontaneous improvement: female sex (p = 0,007) and V377I at the homozygous state (p = 0,0003).

## Conclusion

The homozygous state for V377I and female sex are associated to a spontaneous improvement of disease course in MKD patients. These elements could help clinicians to establish which patients could be exclusively treated with steroid on demand in respect to those that could take advantage from the early use of 2^nd ^line treatment with biologics.

## Disclosure of interest

None declared.

